# Comparison of Pelvic Organ Prolapse Quantification and Simplified Pelvic Organ Prolapse Quantification Systems in Clinical Staging of Iranian Women with Pelvic Organ Prolapse

**DOI:** 10.4314/ejhs.v30i6.10

**Published:** 2020-11

**Authors:** Zinat Ghanbari, Saloumeh Peivandi, Maryam Deldar Pasikhani, Foroohar Darabi

**Affiliations:** 1 Professor, Department of Obstetrics & Gynecology, Faculty of Medicine, Tehran University of Medical Sciences, Tehran, Iran; 2 Assistant Professor, Department of Gynecology and Obstetrics, Faculty of Medicine, Mazandaran University of Medical Sciences, Sari, Iran; 3 Assistant Professor, Department of Obstetrics & Gynecology, Faculty of Medicine, Tehran University of Medical Sciences, Tehran, Iran; 4 Resident of Gynecology and Obstetrics, Faculty of Medicine, Tehran University of Medical Sciences, Tehran, Iran

**Keywords:** Pelvic Organ Prolapse, Observer Variation, POP-Q, Simplified POP

## Abstract

**Background:**

Pelvic organ prolapse is a common pelvic disorder among women. A standard staging system is needed to carefully evaluate the extent and severity of the disease, and initiate appropriate treatment. The aim of this study was to compare the two methods of standard and simplified pelvic organ prolapse quantification systems in clinical staging of Iranian women with pelvic organ prolapse.

**Methods:**

This observational cross-sectional study was conducted on all women with complaints of seeing or feeling a vaginal lump or bulge and/or a dragging sensation who were presented to a pelvic floor disorders clinic of Imam Khomeini Hospital in Tehran, Iran, from October 2018 to June 2019. All patients were evaluated in terms of pelvic organ prolapse severity and staging using both instruments. Also, length of time needed to complete the questionnaires were calculated. After data collection, the results of pelvic organ prolapse staging and degree of agreement between two examiners were evaluated.

**Results:**

A total of 120 women with mean age of 50.92±13.12 years were evaluated. It was shown that there is an almost perfect agreement (kappa coefficient > 0.8) between standard and simplified pelvic organ prolapse quantification systems in all the 3 compartments. Also, there was almost a twofold increase in the time needed to perform standard pelvic organ prolapse quantification (4.16±1.01 minutes) compared to performing simplified pelvic organ prolapse quantification (2.12±1.14 minutes) (p=0.03).

**Conclusion:**

According to the results of this study, there is a substantial and almost perfect agreement between standard and simplified pelvic organ prolapse quantification systems in clinical staging of Iranian women with pelvic organ prolapse. It seems that using simplified pelvic organ prolapse quantification system is more applicable in clinical practice for staging of pelvic organ prolapse, with high reliability coefficient

## Introduction

Pelvic Organ Prolapse (POP) is the movement of one of the pelvic organ (especially bladder, uterus, or rectum) downward and forward relative to its normal location. It is common and progressively affects a large percentage of older women. Although its associated mortality is negligible, it has a significant impact on women's physical, psychological and social wellbeing, which consequently worsens their quality of life (1–3). Therefore, accurate identification of this problem and its aggravating factors is the first step in developing appropriate therapeutic strategies (4–5). Despite numerous studies, the exact cause of POP has not been fully established. However, some potential predisposing factors for POP have been proposed which includes obesity, genetic factors, white female, pregnancy and childbirth, hysterectomy, myopathy and neuropathy (1,6). As long as the prolapsed organ is above the hymen, patients may experience symptoms such as a feeling of pressure or heaviness in the pelvic area, and sometimes pelvic or back pain. Abdominal pain, constipation and urinary symptoms are other possible signs and symptoms of POP (7–9). POP can affect and impair the women's urination, defecation and sexual function. Due to increased life expectancy of women, POP has become a more significant issue that is expected to be doubled in the next 25 years. Therefore, identifying the current status and exact risk factors of POP in women is an important aspect in the prevention and treatment of this disease (7–8). Additionally, the annual cost of POP treatment in the United States is estimated as more than $1 billion, and is expected to increase as the global average ages (9–10).

Despite its importance, there is a limited number of population-based epidemiological studies to evaluate the prevalence of POP, because pelvic examination is a major barrier to these studies (11–13). Performing pelvic examinations in women in a large epidemiological study requires a lot of time, resources and costs, in addition to embarrassment and discomfort of women with clinical examinations, which is a significant limitation for participants in this type of study. In several studies, to overcome these limitations, various questionnaires have been introduced to measure and screen for POP, which lead to different results. In addition, the multiplicity of questions limited the widespread use of these tools for screening (14–18). POP quantification (POP-Q) is the standard and well-known system for evaluating POP, which was designed in 1996 by the American Urological Association and the Urological Surgeons Association (6). This system is very complete and accurate and has the ability to detect abnormalities and disorders even at low stages. Therefore accepted as the gold standard in this field. In the POP-Q system, the test results are standardized and comparable to different centers. The POP-Q system includes 9 special measurements, 6 of which are measured along the vagina and in conjunction with the hymen. In the case of proximal or distal placement relative to the hymen, they are expressed as positive or negative numbers (in centimeters), and the degree of prolapse is determined based on these points (19–20). Although this system has been evaluated and validated in many studies and its effectiveness has been confirmed, it has not yet been able to achieve its success due to many factors such as fertility, difficulty and mismatch between users (low agreement coefficient) and time consumption. Based on this, the International Association of Urology designed a simpler system (simplified POP) based on the same POP-Q system (20–21). Very few previous studies evaluated the association between POP-Q and simplified POP and suggest that these tools should be evaluated in other sociocultural contexts to generalize the results (12,14,18). Considering the high prevalence of pelvic floor dysfunctions among Iranian women (2,6) and the fact that there is no published study to compare these two evaluating systems in Iranian women with POP, this study was conducted to compare POP-Q and simplified POP in clinical staging of Iranian women with POP.

## Methods

This observational cross-sectional study was conducted on all women with complaints of seeing or feeling a vaginal lump or bulge and/or a dragging sensation who were presented to a pelvic floor disorders clinic of Imam Khomeini Hospital in Tehran, Iran, from October 2018 to June 2019. The study protocol was approved by Institutional Ethics Committee (code: IR.TUMS.IKHC.REC.1398.157) and informed consent was obtained from patients.

Patients were selected by simple random sampling. The inclusion criteria were age over 18 and women who complained of vaginal lump or bulge and/or a dragging sensation. Inability to perform the examination due to women's physical disability, pregnancy and history of oophorectomy or hysterectomy were exclusion criteria. Also, women with the previous history of POP surgery were excluded from the study. Patients' demographic and clinical characteristics were collected by a trained obstetrics and gynecology resident. All eligible patients were evaluated in terms of POP severity and staging using both instruments (POP-Q and simplified POP), with empty bladder in lithotomy position. All evaluations were performed separately by two pelvic floor disorders specialists who were blinded to each other's results of POP staging. After data collection, the results of POP staging and degree of agreement between two examiners were evaluated. Also, length of time needed to complete the questionnaires were evaluated and recorded. The POP-Q system consists of measuring 9 defined points and landmarks in centimeter, above (negative number) or below (positive number) to the hymen. Hymen has been considered as reference point. This system classifies POP into 5 stages: stage 0 (no prolapse), stage 1 (most distal portion of the prolapse is more than 1cm above the level of hymen), stage 2 (most distal portion of the prolapse is 1 cm or less proximal or distal to the hymen), stage 3 (most distal portion of the prolapse protrudes more than 1 cm below the hymen) and stage 4 (complete eversion of vaginal walls) (19,22).

Simplified POP is a simpler system that follows the same POP-Q system as the ordinary stage designed by the International Urological Association. In this system, four areas are examined. These include the anterior and posterior walls of the vagina, apex and cervix. In patients with hysterectomy, only three measurements, including anterior and posterior walls of the vagina and cuff (cuff scar/apex) were taken. No measuring devices are required for the simplified POP, and clinicians have to estimate the points at the anterior and posterior parts of the vagina (13–14). This system classifies POP into 4 stages: stage 1 (given point ≥1 cm above hymen), stage 2 (given point descends to introitus from 1 cm above to 1 cm below the hymen), stage 3 (given point ≥1 cm past hymen) and stage 4 (complete vaginal vault eversion/procidentia) (13–14).

**Sample size**: The estimation of sample size was based on a presumed effect size of 0.3 (21), a statistical power of 95%, and a type I error of 5% using G*Power software, version 3.1.3 with the formula for calculation of sample of correlational studies. The overall proper sample size was found to be 120 participants.

**Statistical analysis**: The data's normal distribution was assessed using Kolmogorov-Smirnov statistical test. Mean and standard deviation (SD) as well as frequency (%) were used to display quantitative and qualitative data. Also, chi-squared, student's t-test, Mann-Whitney U and paired t-test were used for data analysis. In order to evaluate the percentage of agreement or the degree of inter-system association, Kappa and Kendall's coefficient of Concordance were used. P-value less than 0.05 was considered as statistically significant.

## Results

A total of 120 patients with a mean age of 50.92 ±13.12 years (range: 29–86) were included in this study. The mean gravidity of the women was 4.87 ± 2.75 (Median: 4; range: 0–13), while the mean Body Mass Index (BMI) of participants was 26.44 ± 2.58 (range: 20–33). Also, the mean duration of completing the questionnaire in POP-Q and simplified POP systems was 13.30±2.50 (median: 13) and 6.50±1.20 minutes (median: 5), respectively. Of the women included in the analyses, 61.7% (74 cases) were post-menopausal and 10% (12 people) had a history of current smoking. Kapa's agreement coefficient is always a number between +1 and -1; +1 means perfect agreement and -1 means perfect disagreement. Based on the results, it was shown that there is an almost perfect agreement between the POP-Q and simplified POP in all the 3 compartments (Table 1).

**Table 1:** Agreement between POP-Q and simplified POP in 3 compartments

Prolapse Stage		Apical Prolapse-Simplified POP				Total	Kappa value	P-value
		I	II	III	IV			
**Apical** **Prolapse-** **POP-Q**	I	76	4	0	0	80	.80	< .001
	II	1	7	5	0	13		
	II	0	0	22	1	22		
	IV	0	0	0	4	4		
Total		77	11	27	5	120		

Prolapse Stage		**Anterior Prolapse- Simplified POP**				**Total**	**Kappa** **value**	**P-value**
		I	II	III	IV			
**Anterior** **Prolapse-** **POP-Q**	I	16	1	0	0	17	.81	< .001
	II	7	63	2	0	72		
	II	0	1	24	1	26		
	IV	0	1	0	4	5		
Total		23	66	26	5	120		

Prolapse Stage		**Posterior Prolapse- Simplified POP**				**Total**	**Kappa** **value**	**P-value**
		**I**	**II**	**III**	**IV**			
**Posterior** **Prolapse-** **POP-Q**	I	46	3	0	0	49	.82	< .001
	II	7	44	0	0	51		
	II	0	1	14	1	16		
	IV	0	0	0	4	4		
Total		53	48	14	5	120		

As shown in Table 2, there is a significant relationship between BMI and prolapse intensity in all the 3 compartments (based on POP-Q method) (P<0.001).

**Table 2:** Relationship between BMI and prolapse intensity in 3 compartments

Prolapse	Apical Prolapse				Anterior Prolapse				Posterior Prolapse				
Stage	N	Mean	SD	P-value	N	Mean	SD	P-value	N	Mean	SD	P-value
**I**	83	25.84	2.38	**<0.001**	16	25.56	2.58	**<0.001**	49	26.08	2.44	**<0.001**
**II**	13	26.54	2.29		72	26.06	2.39		51	25.98	2.32	
**III**	20	27.80	2.11		27	27.19	2.30		16	27.69	2.33	
**IV**	4	31.75	.95		5	30.80	2.28		4	31.75	.95	

Also, the relationship between gravidity and prolapse intensity was statistically significant based on POP-Q method (P <0.05) (Table 3).

**Table 3:** Relationship between gravidity and prolapse intensity in 3 compartments

Prolapse Stage	Apical Prolapse				Anterior Prolapse				Posterior Prolapse			

	N	Mean	SD	P-value	N	Mean	SD	P-value	N	Mean	SD	P-value
**I**	83	4.33	2.22	**0.01**	16	3.50	2.25	**<0.001**	49	4.18	2.34	**0.005**
**II**	13	6.54	3.64		72	4.42	2.20		51	4.78	2.64	
**III**	20	5.75	2.91		27	6.63	3.10		16	6.88	2.57	
**IV**	4	6.25	5.67		5	6.20	4.91		4	6.25	5.67	

The relationship between the type of delivery and the prolapse intensity (based on the POP-Q system) was not significant in any of the compartments (p>0.05). However, there was a significant relationship between menopausal status and prolapse intensity (based on POP-Q system) in all the 3 compartments. Menopausal women had more severe prolapse (Table 4).

**Table 4:** Relationship between menopause and prolapse intensity in 3 compartments

Prolapse Stage	Apical Prolapse			Anterior Prolapse			Posterior Prolapse		

	Menopause	Non-Menopause	P-value	Menopause	Non-Menopause	P-value	Menopause	Non-Menopause	P-value
**I**	23	60	**0.001**	7	9	**0.03**	17	32	**0.01**
**II**	7	6		21	51		16	35	
**III**	12	8		14	13		9	7	
**IV**	4	0		4	1		4	0	

As shown in Table 5, the relationship between smoking status and prolapse intensity was significant in all the 3 compartments. Women who smoked had higher rates of prolapse (P<0.05).

**Table 5:** Relationship between smoking and prolapse intensity in 3 compartments

Prolapse Stage	Apical Prolapse			Anterior Prolapse			Posterior Prolapse		

	Smoking	Non-Smoking	P-value	Smoking	Non-Smoking	P-value	Smoking	Non-Smoking	P-value
**I**	2	81	**0.001**	1	15	**0.006**	2	47	**0.001**
**II**	2	11		4	68		2	49	
**III**	5	15		4	23		5	11	
**IV**	3	1		3	2		3	1	

Also, there was a positive relationship between the women's age and the severity of prolapse in all the 3 compartments (Table 6).

**Table 6:** Relationship between age and prolapse intensity in 3 compartments

Prolapse Stage	Apical Prolapse				Anterior Prolapse				Posterior Prolapse			

	N	Mean	SD	P-value	N	Mean	SD	P-value	N	Mean	SD	P-value
**I**	83	47.10	11.38	**0.001**	16	47.13	10.94	**0.001**	49	49.84	12.62	**0.01**
**II**	13	58.77	13.77		72	47.61	12.13		51	48.24	13.04	
**III**	20	57.70	11.30		27	59.00	11.13		16	57.81	7.85	
**IV**	4	70.75	14.24		5	67.00	14.91		4	70.75	14.24	

The two groups had a statistically significant difference in terms of length of time to complete the questionnaire (P=0.03). There was almost a twofold increase in time needed to perform POP-Q (4.16±1.01 minutes) compared to performing simplified POP (2.12±1.14 minutes).

## Discussion

The results of our study indicate an almost perfect agreement between the POP-Q and simplified POP in clinical staging of POP in all apical, anterior and posterior compartments. As an international standard and reliable system for describing the anatomical position of the pelvic organs, POP-Q system can help physicians to improve the quality of medical care. Despite its well-documented advantages, the perceived difficulties in using this system may initially eliminate the physicians' enthusiasm for its routine clinical use. In response to this concern, a simplified system for evaluating the intensity of POP (Simplified-POP) has been proposed (11). A multi-center study conducted on four continents showed that simplified POP was very well matched to POP-Q (23). The results of this study were consistent with our findings. As shown in our study, the value of Kappa for each of the 3 compartments is greater than 0.8. Therefore, we can use simplified POP with high reliability coefficient instead of POP-Q. The results of our study were in complete agreement with the results obtained from the abovementioned multicenter study. However, the agreement between the two instruments in the present study was higher than that of Swift et al's study (13) that had a moderate to good agreement (0.61) for the anterior and posterior vaginal walls. However, the results of other studies by Manonai et al. (14) and Raizada et al. (12) showed better agreement (0.71 and 0.86, respectively). According to the results of our study, assessing POP using POP-Q needs more time (almost twofold) compared to simplified POP. The POP-Q system consists of measuring 9 specific points, but simplified POP has a much easier use with only four measuring points, with a substantial and almost perfect agreement between these two systems. Parekh et al. (24) showed a near perfect inter-rater agreement of the simplified POP system for overall stages and all points within the system. Also, Singh et al. (25) confirmed a substantial and near perfect agreement between POP-Q and S-POP.

In general, different screening tools have been developed to identify women with pelvic floor muscle weakness, but there is still no consensus on a simple, accurate and reliable tool for identifying women with pelvic floor pain. Tgerstet et al. in Sweden presented a five-item screening test. The sensitivity and specificity of this model in the study population were 92.5% and 94.5%, respectively. However, in a population-based study, the sensitivity decreased to 66.5% (26). Also, the results of studies that used a 4-items questionnaire, developed by WHO, for assessment of POP do not support its accuracy (27–28).

The results of our study indicated that there is a significant relationship between prolapse intensity in all the 3 compartments with BMI, gravidity, menopausal status, cigarette smoking and age. Despite numerous studies, the exact cause of pelvic organ prolapse is not fully elucidated (1,3). However, previous studies have shown that increasing age, obesity and increase in the number of births are the most important factors affecting POP (12–15). In a study by Rodrigues et al., higher BMI, number of pregnancies and births, forceps-assisted deliveries and weight of newborn were risk factors for developing POP in Brazilian women (29). These findings were consistent with the results of the present study. Despite disagreements among researchers that changes that occur during pregnancy or delivery lead to POP, epidemiological studies have suggested that vaginal delivery is the most important risk factor of POP (30–32). However, in our study, no significant relationship was observed between the type of delivery and the severity of prolapse in any of the compartments. This is probably due to the small sample size, especially in subgroups. Also, in line with the previous studies, it was shown that increase in age is a risk factor for the occurrence and severity of POP (16). Our study showed that women who actively smoked had significantly a higher rate of POP. In general, smoking increases the risk of POP by causing tissues hypoxia, hypoestrogenic condition, diabetes, obesity and neuropathy (1,33–34). In conclusion, the results of this study showed that there is a substantial and almost perfect agreement between POP-Q and simplified POP in clinical staging of Iranian women with POP. Considering that simplified POP is a user-friendly and less complicated scoring system for staging of POP than POP-Q, it seems that using S-POP is more applicable in clinical practice for staging of POP, with high reliability coefficient.
